# Genomic Analyses of the Fungus *Paraconiothyrium* sp. Isolated from the Chinese White Wax Scale Insect Reveals Its Symbiotic Character

**DOI:** 10.3390/genes13020338

**Published:** 2022-02-12

**Authors:** Zuo-Yi Fu, Jia-Qi An, Wei Liu, Hong-Ping Zhang, Pu Yang

**Affiliations:** 1 Institute of Highland Forest Science, Chinese Academy of Forestry, Kunming 650224, China; fuzuoyi000@yeah.net (Z.-Y.F.); aws0306@163.com (J.-Q.A.); 1.liuwei.2@163.com (W.L.); 2 Key Laboratory of Breeding and Utilization of Resource Insects of National Forestry and Grassland Administration, Kunming 650224, China; 3 College of Agriculture and Life Sciences, Kunming University, Kunming 650214, China; z2938456055@yeah.net

**Keywords:** Chinese white wax scale insect, fungi, *Paraconiothyrium*, genome sequence, secondary metabolism, cytotoxicity

## Abstract

The Chinese white wax scale, *Ericerus pela*, is an insect native to China. It harbors a variety of microbes. The *Paraconiothyrium* fungus was isolated from *E. pela* and genome sequenced in this study. A fungal cytotoxicity assay was performed on the *Aedes albopictus* cell line C6/36. The assembled *Paraconiothyrium* sp. genome was 39.55 Mb and consisted of 14,174 genes. The coding sequences accounted for 50.75% of the entire genome. Functional pathway analyses showed that *Paraconiothyrium* sp. possesses complete pathways for the biosynthesis of 20 amino acids, 10 of which *E. pela* lacks. It also had complementary genes in the vitamin B groups synthesis pathways. Secondary metabolism prediction showed many gene clusters that produce polyketide. Additionally, a large number of genes associated with ‘reduced virulence’ in the genome were annotated with the Pathogen–Host Interaction database. A total of 651 genes encoding carbohydrate-active enzymes were predicted to be mostly involved in plant polysaccharide degradation. Pan-specific genomic analyses showed that genes unique to *Paraconiothyrium* sp. were enriched in the pathways related to amino acid metabolism and secondary metabolism. GO annotation analysis yielded similar results. The top COG categories were ‘carbohydrate transport and metabolism’, ‘lipid transport and metabolism’, and ‘secondary metabolite biosynthesis, transport and catabolism’. Phylogenetic analyses based on gene family and pan genes showed that *Paraconiothyrium* sp is clustered together with species from the Didymosphaeriaceae family. A multi-locus sequence analysis showed that it converged with the same branch as *P. brasiliense* and they formed one group with fungi from the *Paraconiothyrium* genus. To validate the in vitro toxicity of *Paraconiothyrium* sp., a cytotoxicity assay was performed. The results showed that medium-cultured *Paraconiothyrium* sp. had no harmful effect on cell viability. No toxins were secreted by the fungus during growth. Our results imply that *Paraconiothyrium sp*. may establish a symbiotic relationship with the host to supply complementary nutrition to *E. pela*.

## 1. Introduction

The Chinese white wax scale insect (*Ericerus pela*) is well known for its wax production. White wax secreted by males has high economic value and is widely used in machinery, food, medicine, and other fields [1,2,3]. The white wax is produced by the second-instar male larvae. The males live from about May to August. The lifespan of females is about one year and they produce eggs in the summer. Males and females of *E. pela* parasitize the branches of the Chinese ash tree (*Fraxinus chinensis*) and glossy privet (*Ligustrum lucidum*) for almost their entire lifespan and remain immobile due to the degeneration of their appendages [4,5,6]. *E. pela* display a varied relationship with microorganisms as a result of their sedentary lifestyle [7]. They may also inherit a variety of microorganisms from their ground-dwelling ancestors [8].

In previous studies, we measured the diversity of microorganisms in *E. pela* and found that they house a variety of microbes; we identified 20 phyla from 128 bacterial families and 4 phyla from 48 fungal families [7]. These microorganisms may be transmitted vertically or obtained from the diet or the environment. The bacteria are mainly concentrated in three families: Comamonadaceae, Streptococcaceae, and Rickettsiaceae. The fungi are less abundant and diverse than the bacteria and are mainly concentrated in the Hypocreales, Pleosporales, and Capnodiales orders Studies on scale insect symbiotic microorganisms have primarily focused on bacterial symbionts [9,10]. However, it is unclear whether these fungi play a role in the long parasitic lifespan of *E. pela*.

With the development of high-through sequencing technology, genomic sequencing has provided a robust tool to explore the genetic aspects of the relationships between fungi and insects [11,12,13]. To date, 3413 fungi genomes have been published in GenBank. More than 500 fungi of the three orders mentioned above have been genome sequenced. These data allow for the reliable taxonomic classification of the fungi that *E. pela* host and will also facilitate comparative genomic analyses to reveal the evolution and gene functions of these fungi. Genome sequences also prove information on the complementariness of genes and pathways related to amino acid biosynthesis with the host. Because plant sap supplies unbalanced nutrition to insects, piercing–sucking insects must acquire essential nutrients through nutrition partners [14,15]. In addition, the genome sequences of a large number of insect symbionts have revealed that all of the symbionts have smaller genomes and a higher AT content than free-living relative species, as well as fast evolutionary rates according to their coding genes [16,17].

To understand the genetic information related to the function of the fungi, we cultured fungi from *E. pela* using homogenized eggs in this study. The isolated fungus was genome sequenced, and its toxicity to cells was analyzed. These studies will provide an important foundation for studying the genome character and understanding the relationship between *E. pela* and fungi.

## 2. Materials and Methods

### 2.1. Fungal Culture and Genomic DNA Isolation

*E. pela* eggs were collected and quickly washed with 75% alcohol. The sterilized eggs were homogenized in a 1.5 mL centrifuge tube (Axygen, San Francisco, CA, USA). The homogenate was diluted with sterile water and 50 µL of the diluted homogenate was inoculated onto PDA medium. The medium was incubated at a constant temperature of 30 °C. The culture status was periodically observed; when mycelium growth was observed, the marginal mycelium was promptly transferred to new PDA medium for purification. This process was repeated several times until a pure culture strain was obtained. The strain was used for sequencing.

Genomic DNA was isolated with the BGI Customized Magnetic Plant Genomic DNA Kit (Tiangen Biotech, Beijing, China) according to the manufacturer’s recommended protocol. The DNA concentration was determined using a Qubit fluorometer (Thermo Fisher Scientific, Waltham, MA, USA) and DNA quality was measured using a Nanodrop 2000 spectrophotometer (Thermo Fisher Scientific, Carlsbad, CA, USA). Then, DNA integrity was evaluated using 0.5% agarose gel electrophoresis.

Universal ITS primers [18] were used to amplify the DNA fragments and the amplification results were sequenced and aligned with the ITS sequences in NCBI using BLASTN.

### 2.2. Library Construction and Sequencing

In this study, whole-genome sequencing was performed based on NGS (BGISEQ platform) and SMRT (PacBio Sequel system).

For BGISEQ sequencing, genomic DNA was fragmented using a g-TUBE device (Covaris, MA, USA). Fragments of 300–400 bp were selected with magnetic beads. After DNA purification, dsDNA end repair, 3′ adenylation, and adapter ligation, the templates were amplified by PCR. Then, the amplification products were again selected with magnetic beads. With a splint oligo sequence and ligase, single-stranded circular DNA (ssCir-DNA) was denatured and circularized. With digestion of the linear DNA and qualification using Agilent 2100, a final library containing an insert with a paired-end sequencing length of 150 bp was formed.

The DNBSEQ library was sequenced with the BGISEQ platform through rolling circle amplification to transform ssCir-DNA into DNA nanoballs (DNBs)—nanospheres containing more than 300 copies. Through high-density DNA nanochip technology, the obtained DNBs were added to the holes of the chip mesh with high-density DNA nanochip technology and sequenced by combined probe anchoring polymerization (CPA).

Approximately one microgram of DNA from the strain was used for library construction. The PacBio library was constructed using the SMRTbell Express Template Preparation Kit 2.0 (Pacific Biosciences, Menlo Park, CA, USA). First, DNA samples were sheared using a g-TUBE device and 10–15 Kb fragments were selected using the BluePippin size selection system (Sage Science, Beverly, MA, USA). After the single-stranded overhang was removed, DNA damage and DNA ends were repaired, A-tailing was conducted, and inserts were ligated to adapters (blunt hairpins) to form the SMRTbell library. Ligation products were purified using AMPure PB beads and then pooled. Selection was conducted and inserts less than 10 kb in size were discarded using the BluePippin size selection system (Sage Science, Beverly, MA, USA). Finally, the library was detected with a Qubit DNA HS Assay Kit, and the insert was checked with an Agilent HS DNA Kit (Agilent Technologies, Santa Clara, CA, USA). Sequencing of the SMRTbell library was performed using the Sequel (PacBio) Sequencing Kit 2.0.1 The SMRT bell corresponds to 1 million ZMWs. With the Sequel Binding Kit 3.0 (Pacific Biosciences, Menlo Park, CA, USA), ZMWs loaded with one template and one DNA sample were prepared for sequencing. Sequence information was analyzed through fluorescent signals linked to dNTPs. Subreads obtained from sequencing also contained redundancy, which was removed in the subsequent procedure.

### 2.3. Genome Assembly

Data generated from BGISEQ were filtered to obtain clean data. Reads with a certain proportion of low-quality bases and Ns were filtered, and contaminating duplicates were removed.

For PacBio sequencing, after removal of the adapters several subreads were generated from the same polymerase reads in one ZMW, and subreads less than 1000 bp were trimmed. Then, these subreads were transformed into a circular consensus sequence with enforced consistency (Table S1).

Before genome assembly, the genome size was estimated by K-mer analysis based on BGISEQ data. In the study, we assigned K a value of 15 (Table S2).

Genome assembly and long-read polishing were performed after data polishing. A Falcon (v0.3.0) was used for de novo assembly based on PacBio long reads. Due to the high error rate, preassembly errors still needed to be corrected. First, corrected subreads were obtained through Pbdagcon (https://github.com/PacificBiosciences/pbdagcon, accessed on 20 March 2020) and Falcon (v0.3.0) after assembly with several software programs, such as Celera (v8.3) and Falcon, and the best assembly result remained. Single-base errors were corrected using GATK (v3.8), and the last gaps were filled with pbjelly2 (v15.8.24) after reads with a long insert size were assembled to transform contigs into scaffolds using SSPACE_Basic (v2.0). To further improve assembly accuracy, polishing steps were executed. Initial polishing, which is available for only PacBio long reads, was carried out. Then, the high-accuracy PacBio-corrected assembly was obtained with the help of BGISEQ short reads.

With rudimentary sequencing and subsequent fragment assembly, it was feasible to carry out genome analysis under general conditions. Genome size, gene number, genome characteristics, and assembly statistics, including contig/scaffold (in both number and length), N50, the GC content, and the gap number, were also preserved.

### 2.4. Repetitive Sequences

The assembly results were compared with the transposon sequence database using the de novo method. A database of assembly sequences was established using RepeatMasker software (v4-0-6) and the de novo method [19]. A transposon model was built using RepeatModeler (v2.0.1) based on the database. Transposon prediction was executed using RepeatMasker software (v4-0-6) with the help of the established model. Tandem repeats were predicted using Tandem Repeat Finder (TRF) [20].

### 2.5. Noncoding RNA

The prediction of ncRNA genes (tRNA, rRNA, sRNA, snRNA, and miRNA) was conducted using several software programs. rRNA was predicted ab initio using RNAmmer [21]. The tRNA region and the secondary structure of the tRNA were predicted by tRNAscan-SE (v1.3.1) [22]. sRNA was obtained by aligning sequences with the database using Rfam (v9.1) [23,24].

### 2.6. Gene Prediction

To determine gene location, homology prediction was conducted by comparing the genome sequence with a protein set from several other reference species using GeneWise (v2.20) [25]. With the help of SNAP (v2010-07-28) [26] and GeneMarkES (v4.21) [27], the gene structure and general statistics of the genome regarding functional elements containing introns, exons and CDSs were identified. Specifically, Augustus (v3.2.1) was used for the prediction of protein-coding genes [28].

### 2.7. Gene Annotation

After gene prediction, gene annotation was performed by aligning the predicted protein sequence with Swiss-Prot protein data using BLASTP to assign each gene an annotation of the best match. Predicted proteins were annotated by searching against the NR database. KEGG [29,30,31] annotation was performed through sequence alignment to identify the pathways in which genes might be involved. GO (v07012019) [32] and COG (v11102014) [33] were also used for gene functional annotation in this study.

Fungal databases are essential, especially for identifying the functions of specific genes in the target fungus. Genes involved in the interaction of pathogen and host were analyzed using PHI (v4.6) [34,35]. In addition, CAZy (v201906) [36] was used to identify genes encoding CAZymes, which can damage the cell wall of the host. Additionally, genes that might be related to the transportation of toxic secondary metabolites were searched for in other universal gene annotation databases.

The prediction of secondary metabolites was performed using the online software antiSMASH [37].

### 2.8. Functional Pathway Analysis of E. pela and Paraconiothyrium sp.

We considered plant sap to be deficient in essential amino acids and vitamins for *E. pela* and, combined with *E. pela* genome annotation, aligned the annotated genes of both with the KEGG database-related pathways and constructed metabolic complementary pathway maps.

### 2.9. Core/Pan-Gene Analysis

To explore the functional similarities and differences between the identified genes, an analysis of core/pan-genes among all samples was carried out. Molecular evidence was also obtained to explain the underlying cause for the phenotypic variation. Gene cluster analysis of protein-coding genes from all samples was performed using CD-HIT (v4.6.6) [38], generating final gene clusters that were regarded as pan-gene clusters. The pan-gene clusters were divided into core genes, specific genes, and dispensable genes. The core genes were contained in every strain, and most were essential for the growth of the strain, such as genes related to metabolism and the production of energy. Certain genes, however, were present in only a specific strain to show their unique characteristics or carry out specific metabolic activities. The rest of the genes were dispensable genes. The related species are *Bimuria novae*-*zelandiae* (GenBank: GCA_010015655.1), *Didymosphaeria enalia* (GenBank: GCA_010094045.1), *Karstenula rhodostoma* (GenBank: GCA_010093485.1), *Laburnicola* sp. JP-R-44 (ascomycetes) (GenBank: GCA_009805535.1), *Lentithecium fluviatile* (Genbank: GCA_010405425.1), *Paraphaeosphaeria sporulosa* (GenBank: GCA_001642045.1), and *Paraphaeosphaeria minitans* (GenBank: GCA_009707825.1).

### 2.10. Phylogenetic Tree Was Constructed

A gene family was constructed based on the genes of related species and the target strain, after which gene family identification was carried out.

The genome sequences of the related species downloaded from NCBI were used for reference to analyze gene families. The species used were the same as those mentioned in Section 2.9, but *L. fluviatile* was an outgroup. The identification of gene families was performed by aligning the protein sequences using BLAST, eliminating redundancy using solar, TreeFam [39] gene family clustering treatment of the alignment results using hcluster_sg, converting the protein alignment results into multiple sequence amino acids in the CDS area, and aligning multiple sequences with clustered gene families using Muscle. A phylogenetic tree was constructed based on a module of core/pan genes, gene families, and resequencing data. In this study, with a gene family module, a phylogenetic tree was constructed based on multiple sequence alignment results by adopting the NJ method with TreeBeST (v1.9.2).

In order to verify the results, the three conserved sequences of ITS, LSU, and Tub of 32 species (Table S3) were selected on GenBank for multi-locus sequence analysis with *Paraconiothyrium* sp. [3,18,40]. Multiple sequence alignment was performed for each conserved gene using Muscle (v5.1). Sequence Matrix was used to concatenate the three conserved genes of each species, and the concatenation order was ITS–LSU–Tub. A phylogenetic tree was constructed using the maximum composite likelihood model of neighbor-joining method with a bootstrap value of 1000 using MEGA11.

### 2.11. Synteny Analysis

To detect the evolution of homologous genomes, we performed synteny analysis of *Paraconiothyrium* sp. and the other six species mentioned in Section 2.9 except for *Lentithecium fluviatile* in pairs at the nucleotide and protein levels. The genetic order of the relatives was used as a standard for analysis. Then, the upper and following axes of the linear synteny graph were constructed after the length of both sequences was reduced by the same proportion. According to BLAST, each pair of nucleic acid sequences in the two alignments was marked in the coordinate diagram based on its position information after size reduction at the same proportion. Then, the amino acid levels were compared using the following method. *Paraconiothyrium* sp. was aligned with the relative as a database and the best hit value of each protein as selected. Then, *Paraconiothyrium* sp. was used as a database and other species were aligned with it. The best hits from the two alignments were selected for synteny analysis.

### 2.12. Cytotoxicity Assay

To validate whether *Paraconiothyrium* sp. could secrete toxic materials, the cytotoxicity of secondary metabolites secreted from *Paraconiothyrium* sp. were evaluated in the *Aedes albopictus* cell line C6/36. The fungus was cultured using liquid PDA medium. After replacement of the medium in the test cell with new RPMI 1640 medium (MeilunBio, Dalian, China), 100 μL of the inoculum was inoculated into 96-well plates that had been preincubated for 24 h in an incubator (37 °C). Then, each dilute *Paraconiothyrium* sp. medium was added to each well. Seven gradients were set up; for each, 0, 1, 2, 4, 6, 8, and 10 µL of liquid PDA medium were added. After 24 h of incubation, 10 μL of CCK-8 solution (Proteintech, Wuhan, China) was added to each well. The samples were incubated until the absorbance at 450 nm was approximately 1.0, as measured with a microplate reader (Thermo Fisher Scientific, Waltham, MA, USA). Homogenates of the strains were also assayed for cytotoxicity. DMSO (Sangon, Shanghai, China) was used as a positive control. The volume gradients of DMSO were 0, 1, 2, 4, 6, 8, and 10 µL in each well. The survival rate was calculated using the following formula:

$$\mathrm{Survival}\;\mathrm{Rate}\left(\%\right)=\frac{\mathrm{OD}\left(\mathrm{Negative}\;\mathrm{control}\;\mathrm{group}\right)-\mathrm{OD}\left(\mathrm{Experimental}\;\mathrm{group}\right)}{\mathrm{OD}\left(\mathrm{Negative}\;\mathrm{control}\;\mathrm{group}\right)-\mathrm{OD}\left(\mathrm{Blank}\;\mathrm{control}\;\mathrm{group}\right)}\text{×}100\%$$

## 3. Results

### 3.1. Fungal Culture

**Figure 1:**
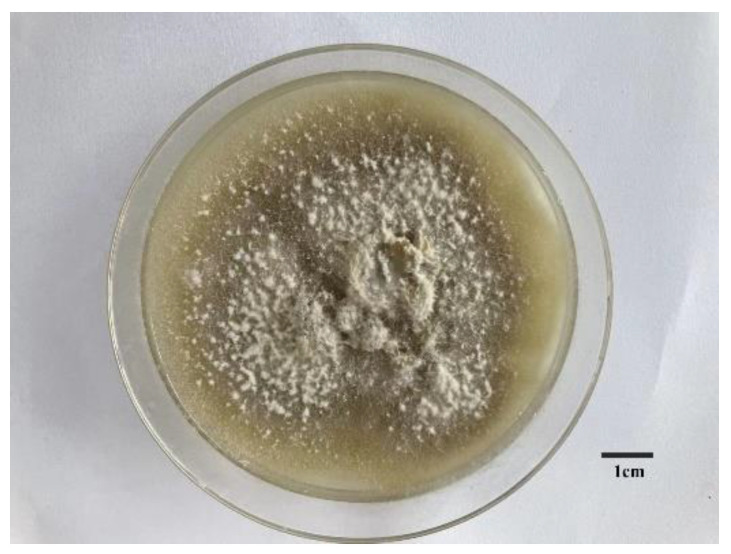
The morphological characters of symbiotic fungus in *Ericerus pela*. Scale bars = 1 cm.

The collected *E. pela* was homogenized and cultured. Two pure fungi were isolated. Internal transcribed spacer (ITS) analyses showed that one fungus exhibits 99.42% similarity with the ITS sequences of *Paraconiothyrium brasiliense*. This fungus was genome sequenced in this study. The morphology of the grown fungus is shown in Figure 1, which exhibits the obvious characteristics of Ascomycetes.

### 3.2. Genome Sequencing and Assembly

**Figure 2:**
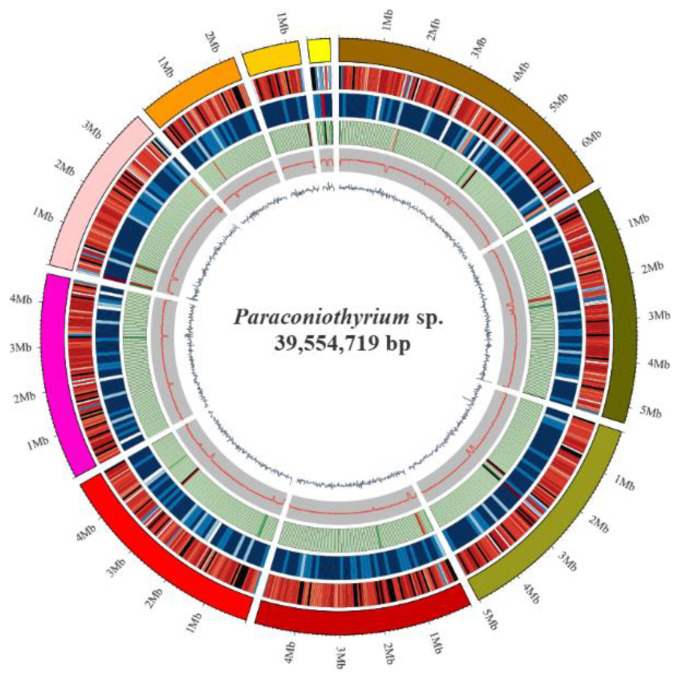
Circular representation of the genomic features of *Paraconiothyrium* sp. From the outer to the inner tracks: Genome Size, Gene density (gene number in 50,000 bp nonoverlapping windows), ncRNA (ncRNA number in 100,000 bp nonoverlapping windows), Repeat coverage (in 50,000 bp nonoverlapping windows), GC rate (in 20,000 bp nonoverlapping windows), GC skew (in 20,000 bp nonoverlapping windows).

The *Paraconiothyrium* sp. genome size was estimated to be approximately 41.79 Mb using K-mer analysis (Figure S1). The *Paraconiothyrium* sp. genome was sequenced using a combination of the BGISEQ and PacBio approaches, and the sequence depths were 86× and 236×, respectively. The assembled *Paraconiothyrium* sp. genome was 39.55 Mb with a scaffold N50 of 4.92 Mb (Figure 2, Table 1 and Table S4). The overall GC content was 51.36% (Table 1).

### 3.3. Genome Components

The *Paraconiothyrium* sp. genome consists of 14,174 genes with an average length of approximately 1.5 kb (Table 1). The CDS accounted for 50.75% of the entire genome with an average length of nearly 1.4 kb, and each gene contained approximately 2.75 exons and 1.75 introns.

Noncoding RNA in *Paraconiothyrium* sp. consisted of tRNA, rRNA, sRNA, snRNA, and miRNA, with copy numbers of 114, 32, 18, 32, and 29, respectively. The average lengths of tRNA, rRNA, sRNA, snRNA, and miRNA were 99.1 bp, 704 bp, 97 bp, 119 bp, and 51 bp, respectively. The total lengths of tRNA, rRNA, sRNA, snRNA, and miRNA were approximately 11.3 kb, 22.5 kb, 1.8 kb, 3.8 kb, and 1.5 kb, respectively (Table S5).

The repetitive sequences were approximately 305, 308, 1124, and 174 kb based on the Repbase database, the ProteinMask database, de novo prediction, and the TRF database, respectively, accounting for 0.77%, 0.78%, 2.84%, and 0.44% of the entire genome, respectively (Table S6). After redundant sequences in the above four databases were removed, 139 kb of repetitive sequences were obtained, accounting for 3.52% of the entire genome. Transposons account for approximately 3.18% of the genome and include DNA (0.16%), LINE (0.06%), LTR (2.46%), SINE (0.01%), and unknown types (0.56%) (Table S7).

### 3.4. Genomic Annotation

GO annotation returned 7238 proteins, accounting for 51.06% of the total proteins (Table 2, Figure S2). These genes were assigned different GO terms. In the biological process category, genes involved in ‘metabolic process’, ‘cellular process’, ‘localization’, ‘biological regulation’, and ‘regulation of biological process’ accounted for the majority. Among the molecular function category, genes were mainly associated with ‘catalytic activity’, ‘binding’, ‘transporter activity’, ‘transcription regulator activity’, and ‘structural molecule activity’.

A total of 1490 classified genes were obtained in 22 clusters of orthologous groups (COG) functional categories (Table 2, Figure S3). Among these categories, except ‘general function prediction only’, the ‘carbohydrate transport and metabolism’ cluster represented the largest group, followed by the ‘lipid transport and metabolism’, ‘amino acid transport and metabolism’, and ‘energy production and conversion’ clusters.

Among all the predicted genes, 4655 (32.84%) genes were mappable through the KEGG pathway database and found to be distributed in 148 metabolic pathways (Table 2, Figure S4). At the third level, these pathways were mainly classified into several categories related to metabolism, such as ‘metabolic pathways’, ‘biosynthesis of secondary metabolites’, ‘microbial metabolism in diverse environments’, ‘biosynthesis of antibiotics’, ‘biosynthesis of amino acids’, and ‘carbon metabolism’.

Secondary metabolism prediction showed many gene clusters that produced polyketide (Table S8).

### 3.5. Analyses of Functional Pathway Related to Nutrition Contribution of Paraconiothyrium sp.

**Figure 3:**
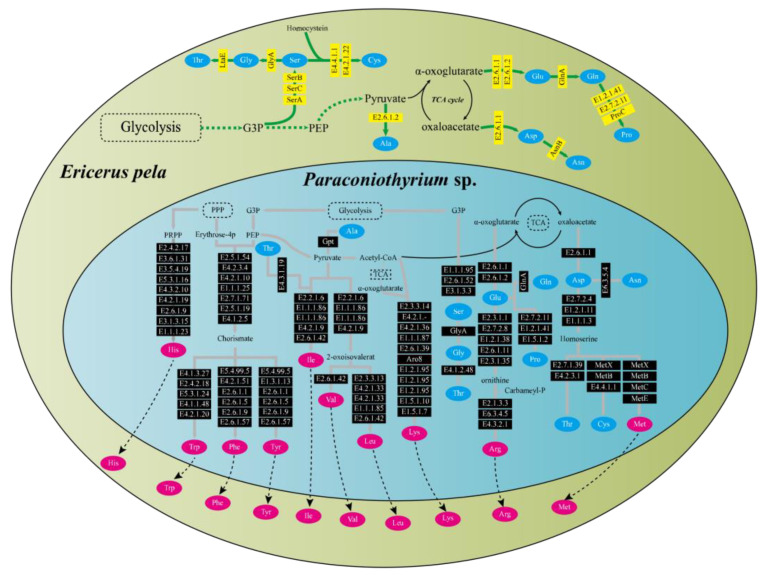
Amino acid synthesis pathways in *E. pela* and *Paraconiothyrium* sp. The green and blue areas represent *E. pela* and *Paraconiothyrium* sp., respectively. The blue and red oval boxes indicate nonessential amino acids and essential amino acid, respectively. Yellow and black boxes represent enzymes whose genes are encoded by *E. pela* and *Paraconiothyrium* sp., respectively. The name of the gene in the box is the EC number or the name of the enzyme corresponding to the KEGG annotation.

Functional pathway analysis showed that *E. pela* and *Paraconiothyrium* sp. were complementary at least in the amino acid synthesis and vitamin synthesis pathways. Genomic annotation indicated that *E. pela* lacked the ability to de novo synthesize 10 essential amino acids (valine, leucine, isoleucine, lysine, arginine, methionine, histidine, phenylalanine, tyrosine, and tryptophan). However, *Paraconiothyrium* sp. has the genes necessary for the synthesis of these essential amino acids (Figure 3). In the vitamin B synthesis pathways, a complementary form of the other is presented. Except for vitamin B6 and riboflavin, which can be synthesized from scratch by *Paraconiothyrium* sp., the complete pathway exists for the other kinds of B vitamins only if the genes of both are complementary (Figure S5).

### 3.6. Identification of Pathogenic Factors

**Figure 4:**
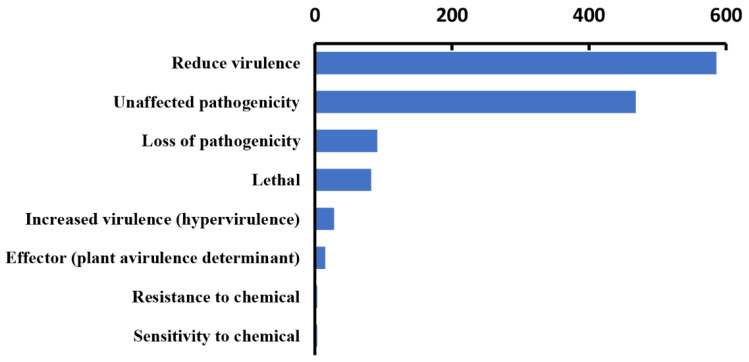
Statistics of virulence-associated genes based on PHI annotation.

In our analysis, 1276 genes with high homology were identified in the PHI database, accounting for 9% of the total number of genes in the *Paraconiothyrium* sp. genome, and they covered 65 fungal species (Table 2). In addition, 540 genes and 511 genes were involved in ’unaffected pathogenicity’ and ’reduced virulence’, respectively. Only 82 genes concern the survival state of the pathogen—‘lethal factor’. In particular, the number of genes strongly associated with ’pathogenicity’ and ‘increased virulence (hypervirulence)’ was only 28 (Figure 4, Table S9).

A total of 595 carbohydrate-active enzyme (CAZyme)-coding gene homologs were identified in the *Paraconiothyrium* sp. genome (Table 2); among these homologs were 261 glycoside hydrolases (GHs), 124 carbohydrate-binding modules (CBMs), 64 glycosyl transferases (GTs), 45 carbohydrate esterases (CEs), 30 polysaccharide lyases (PLs), and 120 enzymes with auxiliary activities (AAs) (Table S10). GHs and AAs were the two most abundant annotated CAZyme genes. Classification of the GH family revealed that the majority of the GHs are members of the GH16 and GH28 families (Table S11). Classification of the AA family showed up to 31 members of AA3, including cellobiose dehydrogenase, glucose 1-oxidase, aryl alcohol oxidase, alcohol oxidase, and pyranose oxidase (Table S11).

### 3.7. Comparative Genome Analyses

**Figure 5:**
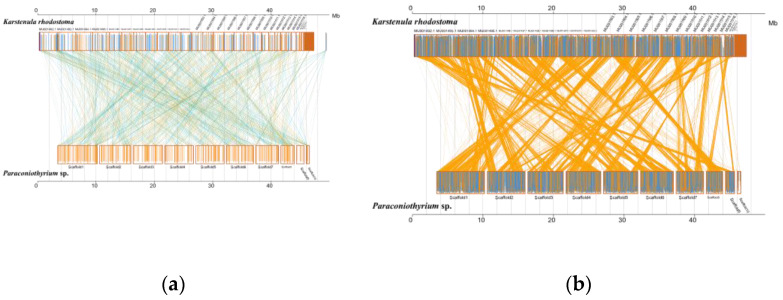
Synteny analysis of *Paraconiothyrium* sp. and *K. rhodostoma* at the nucleic acid and amino acid levels. (**a**) Synteny analysis at the nucleic acid level; (**b**) Synteny analysis at the amino acid level. The yellow box represents the forward chain and the blue box represents the reverse chain within the upper and following sequence regions. In the boxed sequence, the yellow region represents the nucleic acid or amino acid sequence in the forward chain of the genome sequence, and the blue region represents the nucleic acid or amino acid sequence in the reverse chain of the genome sequence. In the region between two sequences, the yellow line represents forward alignment and the blue line represents reverse complementary alignment.

Analysis of MUMmer outputs revealed that the genomes of *K. rhodostoma* (Pleosporales: Didymosphaeriaceae) and *Paraconiothyrium* sp. share 1453 syntenic blocks at the nucleotide level, accounting for approximately 584 kb of the sequence (Figure 5, Table S11). In contrast, *Paraconiothyrium* sp. shares fewer regions of synteny with others (Figure S6, Table S12). This result revealed that *Paraconiothyrium* sp. is more closely related to *K. rhodostoma* than to the other species mentioned in Section 2.9. Similar conclusions were obtained at the protein level (Figure S6, Table S13).

**Figure 6:**
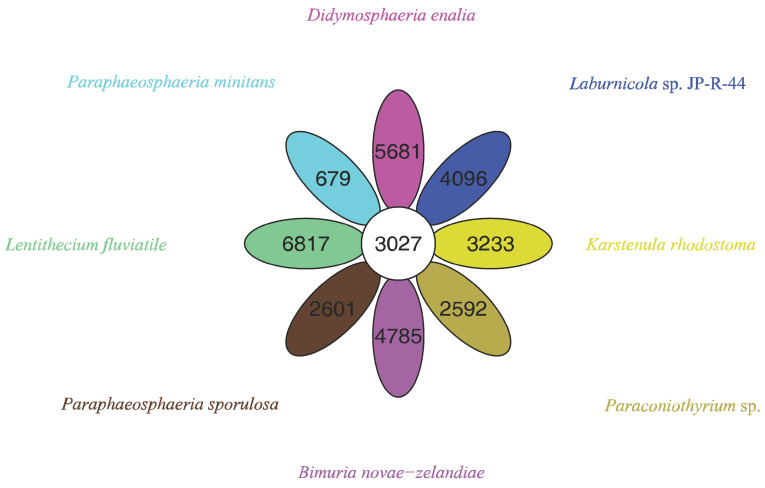
Flower plot based on pan-genomes of the eight species. Each ellipse represents one strain and the number in the ellipse refers to the cluster number. Each cluster has more than 50 percent gene identity and less than 0.3 length diversity.

**Figure 7:**
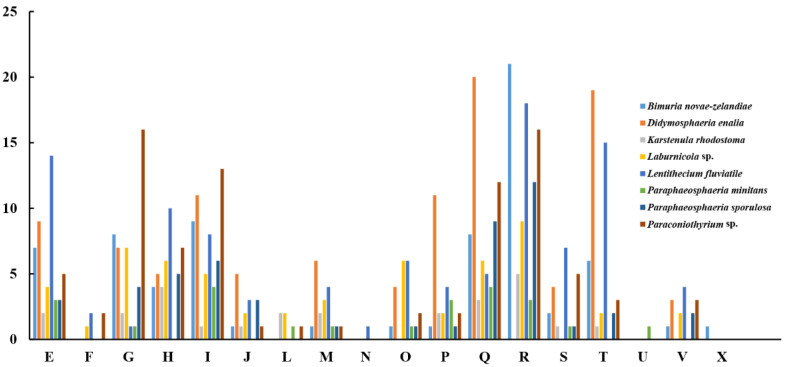
Comparison of COG-based specific genes in eight species. C represents “Energy production and conversion”; E represents “Amino acid transport and metabolism”; F represents “Nucleotide transport and metabolism”; G represents “Carbohydrate transport and metabolism”; H represents “Coenzyme transport and metabolism”; I represents “Lipid transport and metabolism”; J represents “Translation, ribosomal structure and biogenesis”; L represents “Replication, recombination and repair”; M represents “Cell wall/membrane/envelope biogenesis”; N represents “Cell motility”; O represents “Posttranslational modification, protein turnover, chaperones”; P represents “Inorganic ion transport and metabolism”; Q represents “Secondary metabolite biosynthesis, transport, and catabolism”; R represents “General function prediction only”; S represents “Function unknown”; T represents “Signal transduction mechanisms”; U represents “Intracellular trafficking, secretion, and vesicular transport”; V represents “Defense mechanisms”; and X represents “Mobilome: prophages, transposons”.

The pan-genome from eight species from Pleosporales contains 44,480 genes (Table S14). The core genome consists of 3027 genes (Figure 6). Except for *P. minitans*, the percentage of unique genes to the number of coding genes was the smallest for *Paraconiothyrium* sp. Additionally, the proportion of dispensable genes was large among the eight species. *Paraconiothyrium* sp. possesses 2592 specific genes, most of which are concentrated in metabolism-related processes. The top three COG categories were ‘carbohydrate transport and metabolism’, ‘lipid transport and metabolism’, and ‘secondary metabolite biosynthesis, transport, and catabolism’ (Figure 7). Meanwhile, the GO annotation of unique genes revealed genes enriched in the metabolic pathways of secondary products, such as the austinol metabolic process, dehydroaustinol metabolic process, and toxin metabolic process (Table S15).

The phylogenetic tree was constructed using *L. fluviatile* (Pleosporales: Lentitheciaceae) as an outgroup and eight species based on the pan-genome core genes or single-copy ortholog genes, respectively. The topology of the two trees is the same, but the divergence of bases differs slightly. The clusters composed of *K. rhodostoma*, *P. minitans*, and *P. sporulosa* are sister groups to *Paraconiothyrium* sp. (Figure S7). The species of these branches belongs to the Didymosphaeriaceae family.

**Figure 8:**
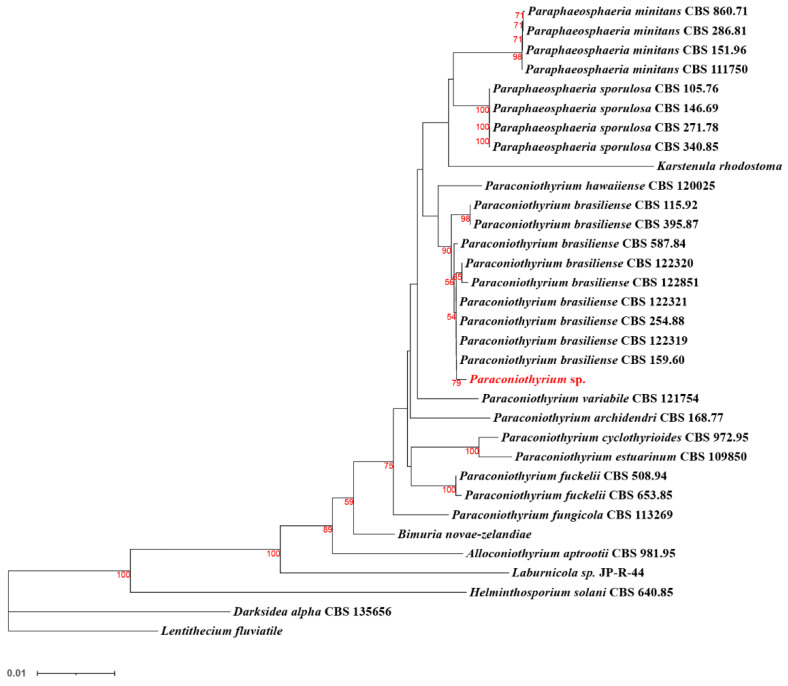
Phylogenetic tree of *Paraconiothyrium* sp. and 32 other species based on the neighbor-joining method. Bolds are the species involved in the phylogenetic analysis. The number below the branch indicates bootstrap values obtained from 1000 replications. Red numbers on the branch nodes indicate bootstrap values. Only bootstrap values equal to or greater than 50 are listed on the branches. The scale bar indicates the genetic distances.

Further multi-locus sequence analysis (Figure 8) showed that *Paraconiothyrium* sp. and *Paraconiothyrium brasiliense* converged upon the same branch. They clustered together with all the species from the *Paraconiothyrium* genus and formed one group in the phylogenetic tree.

### 3.8. Cytotoxicity Assay

**Figure 9:**
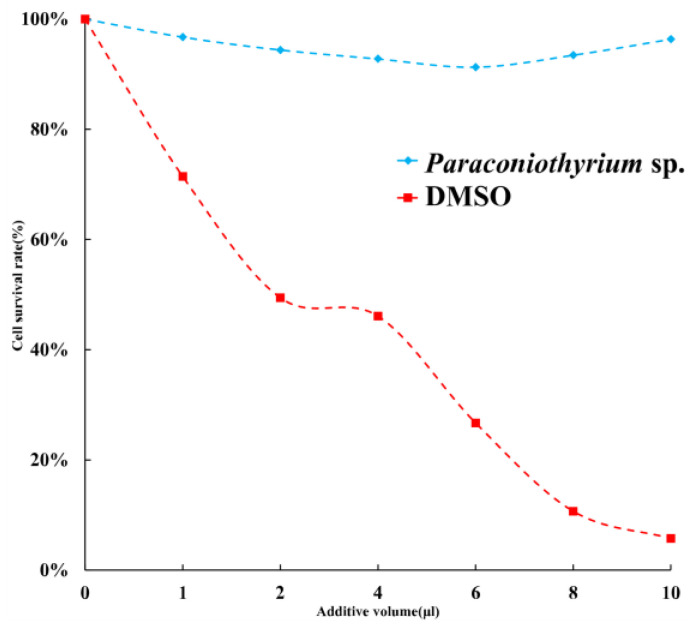
Cell survival rate with different additions. Blue indicates that the additive is PDA liquid medium containing *Paraconiothyrium* sp. metabolites. Red indicates that the additive is DMSO, which was used as a positive control. X-axis indicates the different volumes added, from left to right, 0, 1, 2, 4, 6, 8, and 10 µL. Y-axis indicates the cell survival rate, where the survival rate without the addition is 100%.

To estimate the toxicity of materials secreted by *Paraconiothyrium* sp, the cytotoxicity assay was performed. The results of a cytotoxicity assay showed slight inhibition of the C6/36 cell line by the PDA liquid medium containing secretions. The cell viability was over 90% with different doses of medium. Cell survival was the lowest when 6 µL of medium was added, at approximately 91.3%. We used DMSO, which is highly cytotoxic, as a positive control, and the results showed that the more DMSO was added, the lower the cell survival rate was (Figure 9).

## 4. Discussion

*Paraconiothyrium* is widely distributed and has a variety of host habitats [41]. It has been found living within insects. It was reported that *P. brasiliense* was isolated from *Acrida cinerea* [42]. Another fungus from the *Paraconiothyrium* genus, *P. hawaiiense*, was isolated from the scale insect *Diaspidiotus* sp. [43]. We also found ITS sequences of *Paraconiothyrium* in the microorganism diversity analyses in our previous study [7]. They exist in insects, suggesting that they may play some role in the insect host. We isolated *Paraconiothyrium* sp. From *E. pela* and sequenced the genome in this study. Phylogenetic trees based on gene families showed that *Paraconiothyrium* sp. is evolutionally close to *K. rhodostoma*, *P. minitans*, and *P. sporulosa*.

The genome assembly is approximately 39.55 Mb and smaller than the genomes of the relatives of Didymosphaeriaceae. For example, *B. novae-zelandiae,* the species with the largest genome in the phylogenetic analysis, has a genome size of 78.18 Mb, and the *Paraconiothyrium* sp. genome is reduced in size by nearly half. However, the genomes of obligate symbiotic bacteria range from 0.5–2 Mb. In contrast, the genomes of related free-living bacteria are approximately five times larger (usually 4–10 Mb) [44].

The *P. sporulosa* genome has a GC content of 53.3%, higher than that of the majority of the fungi used for phylogeny. The GC content of the *Paraconiothyrium* sp. genome was 51.36%, similar to that of the *P. sporulosa* genome. However, the GC content of the obligate symbiotic bacterial genome was much lower than that of its free-living relatives [45,46,47,48]. This suggests that *Paraconiothyrium* sp. represents a transition stage between endogenous fungi and obligate symbionts and that the symbiotic relationship between *Paraconiothyrium* sp. and *E. pela* is relatively casual.

By synteny analysis, we found some sequence fragments that are lacking in the *Paraconiothyrium* sp. genome. *E. pela* is a scale insect. According to Gullan, scale insects may have long ago inherited mutual relationships with a variety of microorganisms from their ancestors due to their lifestyles [11]. After establishing a symbiotic relationship with the host, the symbiont becomes host-dependent due to the loss of genes involved in some biological processes [49,50]. Although they lose many genes, symbionts retain certain genes required for nutrient synthesis that are somewhat complementary to the missing parts of the host [51,52]. Additionally, genome annotation of *Paraconiothyrium* sp. showed retention of an intact essential amino acid synthesis pathway and that *Paraconiothyrium* sp. is also capable of producing nonessential amino acids, which should compensate for the host in this regard (Figure 3). In addition, *E. pela* lacks the complete vitamin B synthesis pathway, as shown by the gene functional annotation of the previously reported genome sequence [2]. However, *Paraconiothyrium* sp. contains synthetic pathways for vitamin B group members. *Paraconiothyrium* sp. and *E. pela* may collaborate in the production of most of the vitamin B group members (Figure S5). The above shows that *Paraconiothyrium* sp. plays a significant role in providing nutritional metabolic assistance to the host.

Fungi of the *Paraconiothyrium* genus have often been considered pathogens to plants [41,53]. They can infect the leaves of plants and cause leaf spots. They also cause human cutaneous phaeohyphomycosis [54,55]. However, they have been found living inside scale insects without causing disease, implying their adaptation to an insect host [41,42]. PHI annotation of the *Paraconiothyrium* sp. genome showed genes associated with reduced virulence and unaffected pathogenicity to be common in *Paraconiothyrium* sp. To verify this prediction, we performed a cytotoxicity assay. The *Paraconiothyrium* sp. liquid medium containing secretions had no significant inhibitory effect against the C6/36 cell line. In contrast, DMOS was substantially toxic to the cells. The presence of a symbiotic relationship between *Paraconiothyrium* sp. and *E. pela* can be inferred from this finding.

Evidence suggests that certain symbionts can assist the host in penetrating plant cell walls [47,48,56]. A large number of GH genes, such as GH16, GH28, GH43, and GH47, have also been found in the *Paraconiothyrium* sp. genome, and their main role involves the degradation of plant cell wall hemicellulose and pectin, suggesting that *Paraconiothyrium* sp. can assist *E. pela* in piercing plant tissues.

Lignocellulose-degrading fungi usually contain genes encoding lytic polysaccharide monooxygenases (LPMOs) [57], which are classified in the AA family in the CAZyme database. These enzymes are mainly involved in the depolymerization of lignin [58]. Thirty-one AA3 and 37 AA9 family genes were found in the *Paraconiothyrium* sp. genome. Members of the AA3 family assist other AA family enzymes through hydrogen peroxide or hydroquinone or assists glycoside hydrolases in the degradation of lignocellulose. LPMOs, which belong to the AA9 family, are involved in the degradation of various hemicelluloses from cellulose and lignocellulose, such as xyloglucan, xylan, and glucomannan [58]. A large number of AA3 and AA9 genes work together to degrade lignin by oxidation [58]. Experiments have shown that *Paraconiothyrium* sp. is highly abundant in the host epidermis and digestive tract. Both families are hypothesized to assist in digestion or in the invasion of plants by the insect through epidermal contact. These AAs facilitate the establishment of a symbiotic relationship between *E. pela* and *Paraconiothyrium* sp.

## Figures and Tables

**Table 1:** Main assembly features of *Paraconiothyrium* sp.

Assembly Features and Genome Features	Value
Total genome size (Mb)	39.55
Max Length (bp)	6,769,356
N50 (bp)	4,916,146
N90 (bp)	2,311,653
GC (%)	51.36
Number of genes	14,174
Number of exons	38,925
Number of CDS	14,174
Number of introns	24,751
Gene length/genome length (%)	4.43
Exons length/genome length (%)	55.61
CDS length/genome length (%)	50.75
Intron length/genome length (%)	4.86
Average gene length (bp)	1552
Average exon length (bp)	515.7
Average CDS length (bp)	1416
Average intron length (bp)	77.7
tRNA	114

**Table 2:** Statistics of annotation of different databases.

	Database	Number	Percent
Annotated	P450	2436	17.18%
	CAZY	595	4.19%
	KINASE	141	0.99%
	SWISSPROT	3531	24.91%
	NOG	9248	65.24%
	COG	1490	10.51%
	CARD	2	0.01%
	NR	12,411	87.56%
	DBCAN	782	5.51%
	TCDB	620	4.37%
	IPR	10,220	72.10%
	PHI	1276	9%
	KEGG	4655	32.84%
	GO	7238	51.06%
	PHOSPHATASE	35	0.24%
	KOG	2201	15.52%
Unannotated	-	1342	9.47%
Total	-	12,832	90.53%

## Data Availability

The data is available from GenBank repository with accession number JAJUBI000000000, and BioProject number PRJNA791143.

## Supplementary Materials

The following supporting material can be downloaded at: https://www.mdpi.com/article/10.3390/genes13020338/s1, Figure S1. 15-mer analysis. X-axis is depth, and Y-axis is proportion. Theoretically, 15-mer distributions should follow a Poisson distribution. In fact, heterozygotes cause the possible appearance of other peaks at 1/2 of the main peak, and duplication causes the possible appearance of duplicate peaks near integer multiples of the main peak. Figure S2. Functional categories of the annotated genes, broadly separated into ‘biological process’, ‘cellular component’ and ‘molecular function’ based on Gene Ontology. X-axis indicates 45 functional GO categories. Blue boxes represent biological processes, yellow boxes represent cell composition, and orange boxes represent molecular functions. Y-axis indicates number of genes in a category. Figure S3. Cluster of orthologous groups (COG) classification of putative proteins. Y-axis indicates 22 functional COG categories. X-axis indicates number of genes in a category. Figure S4. KEGG classification of the genes. A total of 4655 genes were assigned to 383 KEGG pathways. X-axis indicates number of genes in a pathway. Y-axis indicates 46 second level pathways. Figure S5. Pathways of vitamin B synthesis involving *E. pela* and *Paraconiothyrium* sp. Red boxes and lines indicate genes and pathways unique to *Paraconiothyrium* sp. Green boxes and lines indicate genes and pathways unique to *E. pela*. Gray dashed lines and boxes indicate that neither gene or pathway is present. Figure S6. Synteny analysis between *Paraconiothyrium* sp. and the other species at the nucleic acid and amino acid level. Yellow box stands for forward chain and blue box stands for reverse chain within the upper and following sequence region. In the box of sequence, the yellow region stands for the nucleic acid sequence in the forward chain of this genome sequence and the blue region stands for the nucleic acid sequence in the reverse chain of this genome sequence. In the middle region of two sequences, the yellow line stands for forward alignment and the blue line stands for reverse complementary alignment. Column A is based on nucleotide level, column B is based on amino acid level. The species analyzed from top to bottom with *Paraconiothyrium* sp. are *Bimuria novae-zelandiae*, *Didymosphaeria enalia*, *Laburnicola* sp. JP-R-44, *Paraphaeosphaeria sporulosa* and *Paraphaeosphaeria minitans*. Figure S7. Phylogenetic tree in the eight species determined by the maximum likelihood method. *L. fluviatile* is the outgroup. (a) based on the pan-genome; (b) based on single-copy ortholog genes. The scale bar corresponds to 0.06 nucleotide substitutions per two sites; Table S1. PacBio reads statistics. Table S2. The statistics of BGISEQ based on next generation sequencing. Table S3. Three conserved sequences used in phylogenetic analyses. Table S4. Genome assembly of *Paraconiothyrium* sp. Table S5. Statistics of noncoding RNA in *Paraconiothyrium* sp. genome. Table S6. Repeat statistic in *Paraconiothyrium* sp. genome. Table S7. Transposons statistic in *Paraconiothyrium* sp. genome. Table S8. Predictions of secondary metabolite using antiSMASH. Table S9. Identification of pathogenicity genes through querying the *Paraconiothyrium* sp. genome with the PHI database. Table S10. Results of classification and annotation of carbohydrate enzymes. CBM, carbohydrate-binding module; CE, carbohydrate esterases; GH, glycoside hydrolases; GT, glycosyl transferase; PL, polysaccharide lyase; AA, auxiliary activity. Table S11. Statistics of all classifications in CAZy database. Table S12. Statistics for synteny at the nucleic acid level. Scaffold represents the 10 scoffolds assembled by *Paraconiothyrium* sp. Refs Length represents the length of each scoffold. Map number and map length represent the genome of the species corresponding to one of the Paraconiothyrium sp. scaffold gene and length, respectively. Table S13.Statistics for synteny at the amino acid level. Aligned indicates the number of genes of *Paraconiothyrium* sp. on the corresponding species match. Target percent (%) represents the number of genes of the corresponding species match as a percentage of *Paraconiothyrium* sp. Query percent (%) represents the number of genes of the corresponding substance match as a percentage of the total number of own genes. Table S14. The statistics of pangenome.
