# Supplementary material for: Genomic Analyses of the Fungus Paraconiothyrium sp. Isolated from the Chinese White Wax Scale Insect Reveals Its Symbiotic Character
Source: Genes (Basel). 2022 Feb 12;13(2):338. doi: 10.3390/genes13020338 (PMC8872350; doi:10.3390/genes13020338)
Supplement: Supplementary file 1 [file genes-13-00338-s001.zip › supplementary figure legends.pdf]

Figure S1. 15-mer analysis. X-axis is depth, and Y-axis is proportion. Theoretically, 15-mer distributions should follow a Poisson distribution. In fact, heterozygotes cause the possible appearance of other peaks at 1/2 of the main peak, and duplication causes the possible appearance of duplicate peaks near integer multiples of the main peak.

Figure S2. Functional categories of the annotated genes, broadly separated into 'biological process', 'cellular component' and 'molecular function' based on Gene Ontology. X-axis indicates 45 functional GO categories. Blue boxes represent biological processes, yellow boxes represent cell composition, and orange boxes represent molecular functions. Y-axis indicates number of genes in a category.

Figure S3. Cluster of orthologous groups (COG) classification of putative proteins. Y-axis indicates 22 functional COG categories. X-axis indicates number of genes in a category.

Figure S4. KEGG classification of the genes. A total of 4,655 genes were assigned to 383 KEGG pathways. X-axis indicates number of genes in a pathway. Y-axis indicates 46 second level pathways.

Figure S5. Pathways of vitamin B synthesis involving *E. pela* and *Paraconiothyrium* sp. Red boxes and lines indicate genes and pathways unique to *Paraconiothyrium* sp. Green boxes and lines indicate genes and pathways unique to *E. pela.* Gray dashed lines and boxes indicate that neither gene or pathway is present.

Figure S6. Synteny analysis between *Paraconiothyrium* sp. and the other species at the nucleic acid and amino acid level. Yellow box stands for forward chain and blue box stands for reverse chain within the upper and following sequence region. In the box of sequence, the yellow region stands for the nucleic acid sequence in the forward chain of this genome sequence and the blue region stands for the nucleic acid sequence in the reverse chain of this genome sequence. In the middle region of two sequences, the yellow line stands for forward alignment and the blue line stands for reverse complementary alignment. Column A is based on nucleotide level, column B is based on amino acid level. The species analyzed from top to bottom with *Paraconiothyrium* sp. are *Bimuria novae-zelandiae, Didymosphaeria enalia*, *Laburnicola* sp. JP-R-44, *Paraphaeosphaeria sporulosa* and *Paraphaeosphaeria minitans*.

Figure S7. Phylogenetic tree in the eight species determined by the maximum likelihood method. *L. fluviatile* is the outgroup. (**a**) based on the pan-genome; (**b**) based on single-copy ortholog genes. The scale bar corresponds to 0.06 nucleotide substitutions per two sites.
