# Supplementary material for: Genomic Analyses of the Fungus Paraconiothyrium sp. Isolated from the Chinese White Wax Scale Insect Reveals Its Symbiotic Character
Source: Genes (Basel). 2022 Feb 12;13(2):338. doi: 10.3390/genes13020338 (PMC8872350; doi:10.3390/genes13020338)
Supplement: Supplementary file 1 [file genes-13-00338-s001.zip › Table S1,S2,S4,S5,S6,S7,S10,S14.pdf]

Table S1. PacBio Reads Statistics

Valid ZWM Number (#)	Subreads Number (#)	Subreads Total Bases (bp)	Subreads Mean Length (bp)	Subreads N50 (bp)	Subreads N90 (bp)	Subreads Max Length (bp)	Subreads Min Length (bp)
124319	867099	9371760881	10808	12096	7535	210638	1,000

Note: Valid ZWM Number, the number of valid ZMWs; Subreads Number, the number of Subreads after filtering; Subreads Total Bases, data size of all Subreads; Subreads Mean Length, the average length of Subreads.

Table S2. The statistics of BGISEQ based on next generation sequencing

Insert Size (bp)	Reads Length (bp)	Raw Data (Mb)	Adapter (%)	Duplication (%)	Total Reads (#)	Filtered Reads (%)	Low Quality Filtered Reads (%)	Clean Data (Mb)
300-400	150:150	3,907	0.98	0.21	26,048,018	12	10.8	3,438

Note: Insert Size, the length of inserted fragment; Reads Length， length of reads; Raw Data， the size of raw data; Adapter， The proportion of Adapter; Duplicaiton， The proportion of same reads; Total Reads， total reads number; Filtered Reads， The proportion of filtered reads; Low Quality Filtered Reads, The proportion of Low quality filtered reads; Clean Data， the size of reads we delivered.

Table S4. Genome assembly of *Paraconiothyrium* sp.

Seq Type	Total Number	Total Length(bp)	N50 Length(bp)	N90 Length(bp)	Max Length(bp)	Min Length(bp)	Gap Number
Scaffold	10	39554719	4916146	2311653	6769356	504433	9022
Contig	14	39545697	4643821	2311653	5362165	9102	-

Note: Seq Type, Contig Or Scaffold; Total Number, number of contig or scaffold; Total Length, the length of whole assembly sequence.

Table S5. Statistics of noncoding RNA in *Paraconiothyrium* sp. genome.

Type	Copy Number	Average length (bp)	Total length (bp)	Percent in Genome (%)
tRNA	114	99.1	11,298	0.0286
rRNA	32	704.18	22,534	0.0570
sRNA	18	97.33	1,752	0.0044
snRNA	32	119.34	3,819	0.0097
miRNA	29	51	1,479	0.0037

Table S6. Repeat statistic in *Paraconiothyrium* sp. genome.

Method	Repeat Size (bp)	% in Genome
Repbase	305015	0.7711
ProMask	307679	0.7779
de novo	1124218	2.8422
TRF	174232	0.4405
Total	1390941	3.5165

Table S7. Transposons statistic in *Paraconiothyrium* sp. genome.

	Repbase TEs		ProteinMask TEs		*de novo* TEs		Combined TEs	
Type	Length (bp)	% in Genome	Length (bp)	% in Genome	Length (bp)	% in Genome	Length (bp)	% in Genome
DNA	33670	0.0851	20760	0.0525	9047	0.0229	63062	0.1594
LINE	19765	0.0500	3134	0.0079	0	0	21859	0.0533
LTR	252056	0.6372	284160	0.7184	906534	2.2918	973176	2.4603
SINE	2518	0.0064	0	0	0	0	2518	0.0064
Other	0	0	0	0	0	0	0	0
Unknown	363	0.0009	0	0	222138	0.5616	222501	0.5625
Total	305015	0.7711	307679	0.7779	1124218	2.8422	1258688	3.1821

Table S10. Results of classification and annotation of carbohydrate enzymes. CBM, carbohydrate-binding module; CE, carbohydrate esterases; GH, glycoside hydrolases; GT, glycosyl transferase; PL, polysaccharide lyase; AA, auxiliary activity.

AAs Number	CBMs Number	CEs Number	GHs Number	GTs Number	PLs Number
120	124	45	261	64	30

Table S14. The statistics of pan genome.

Core Gene Number	Core Gene Size (bp)	Pan Gene Num (#)	Pan Gene Size (bp)	Dispensable Number	Dispensable Size (bp)
3,027	1,832,598	44,480	17,531,546	10,969	5,994,990
