# Supplementary figures and images for: Genomic Analyses of the Fungus Paraconiothyrium sp. Isolated from the Chinese White Wax Scale Insect Reveals Its Symbiotic Character
Source: Genes (Basel). 2022 Feb 12;13(2):338. doi: 10.3390/genes13020338 (PMC8872350; doi:10.3390/genes13020338)

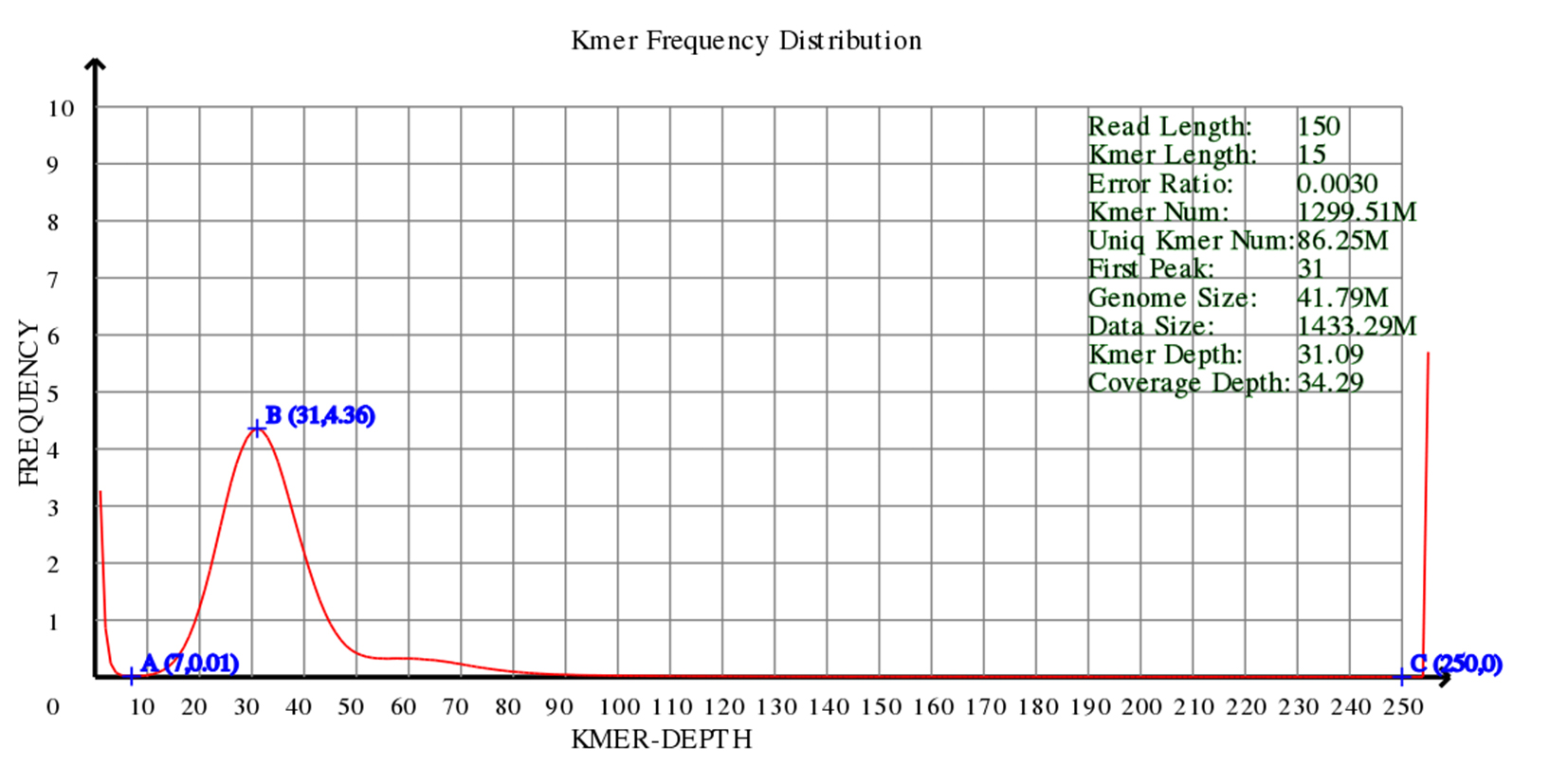

Supplement: Supplementary file 1 [file genes-13-00338-s001.zip › Figure S1.jpg]

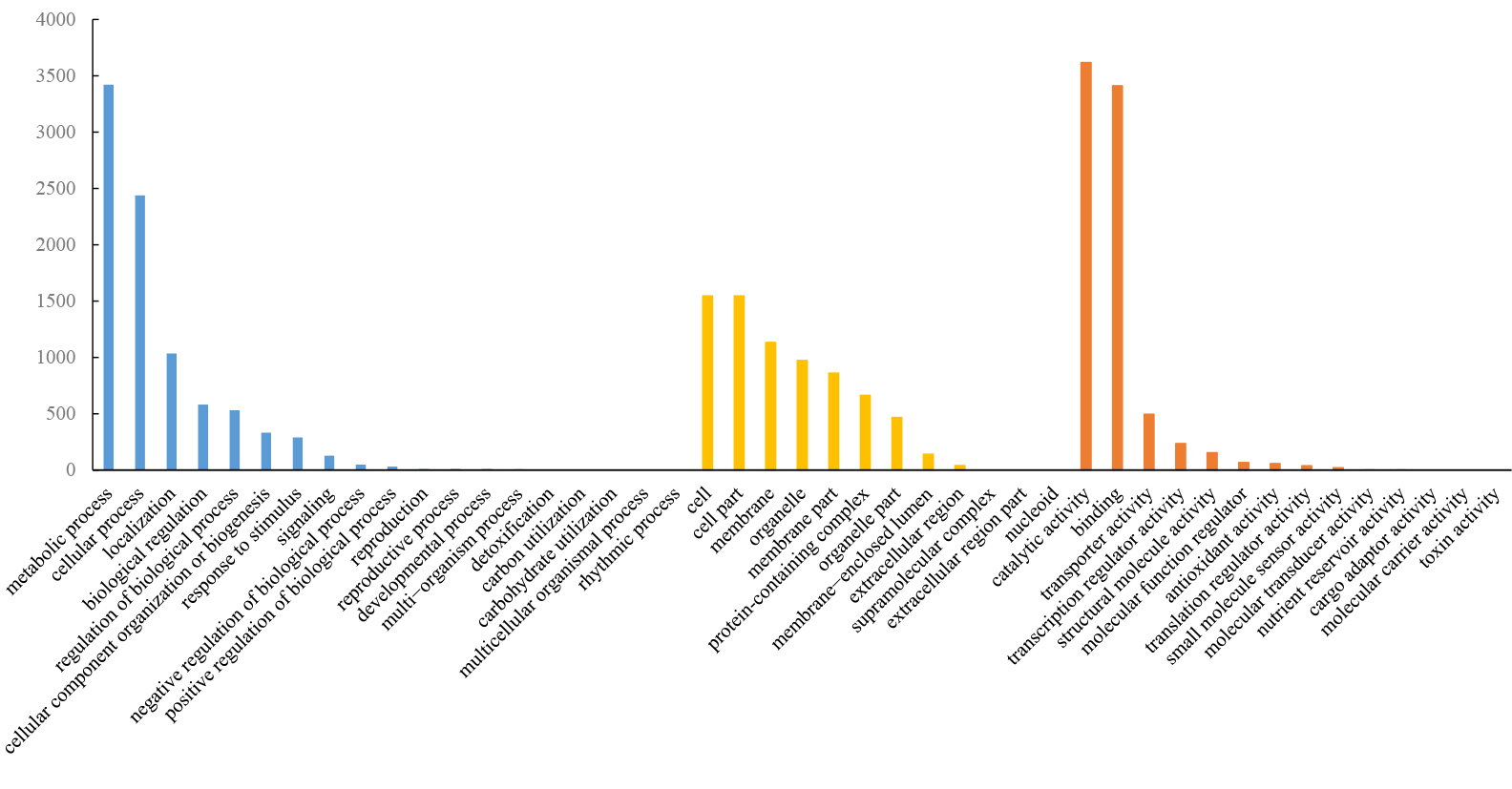

Supplement: Supplementary file 1 [file genes-13-00338-s001.zip › Figure S2.jpg]

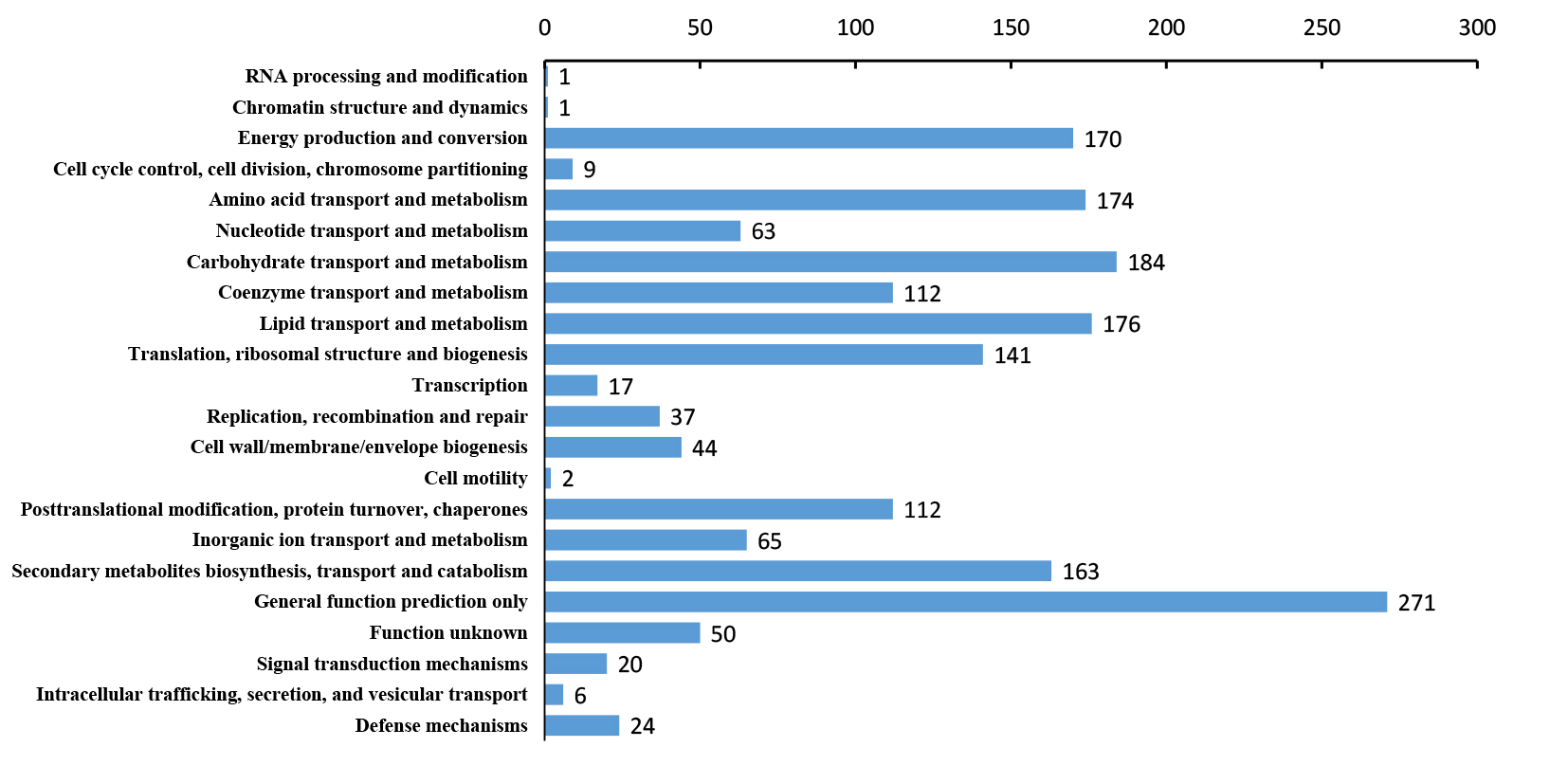

Supplement: Supplementary file 1 [file genes-13-00338-s001.zip › Figure S3.jpg]

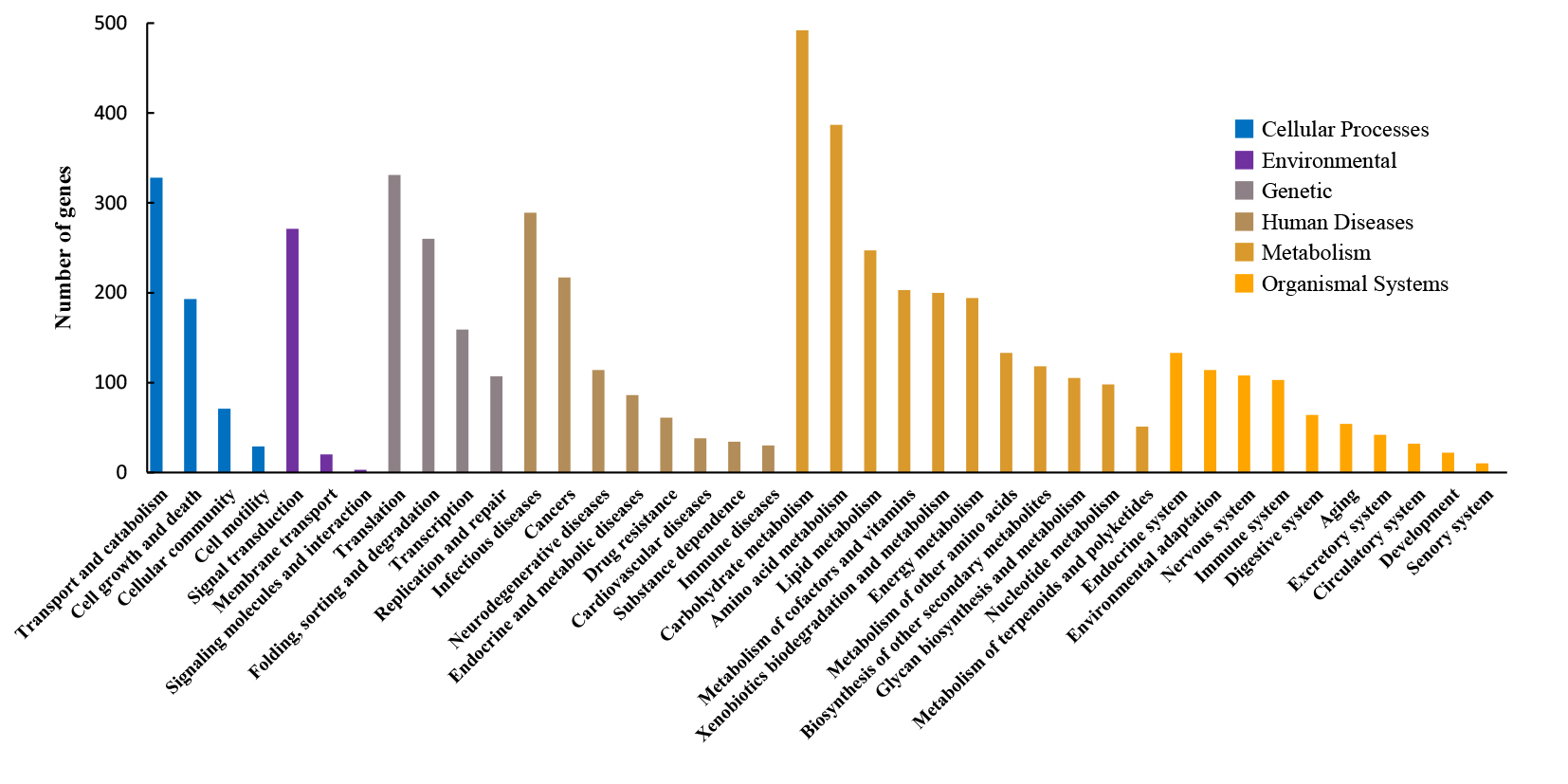

Supplement: Supplementary file 1 [file genes-13-00338-s001.zip › Figure S4.jpg]

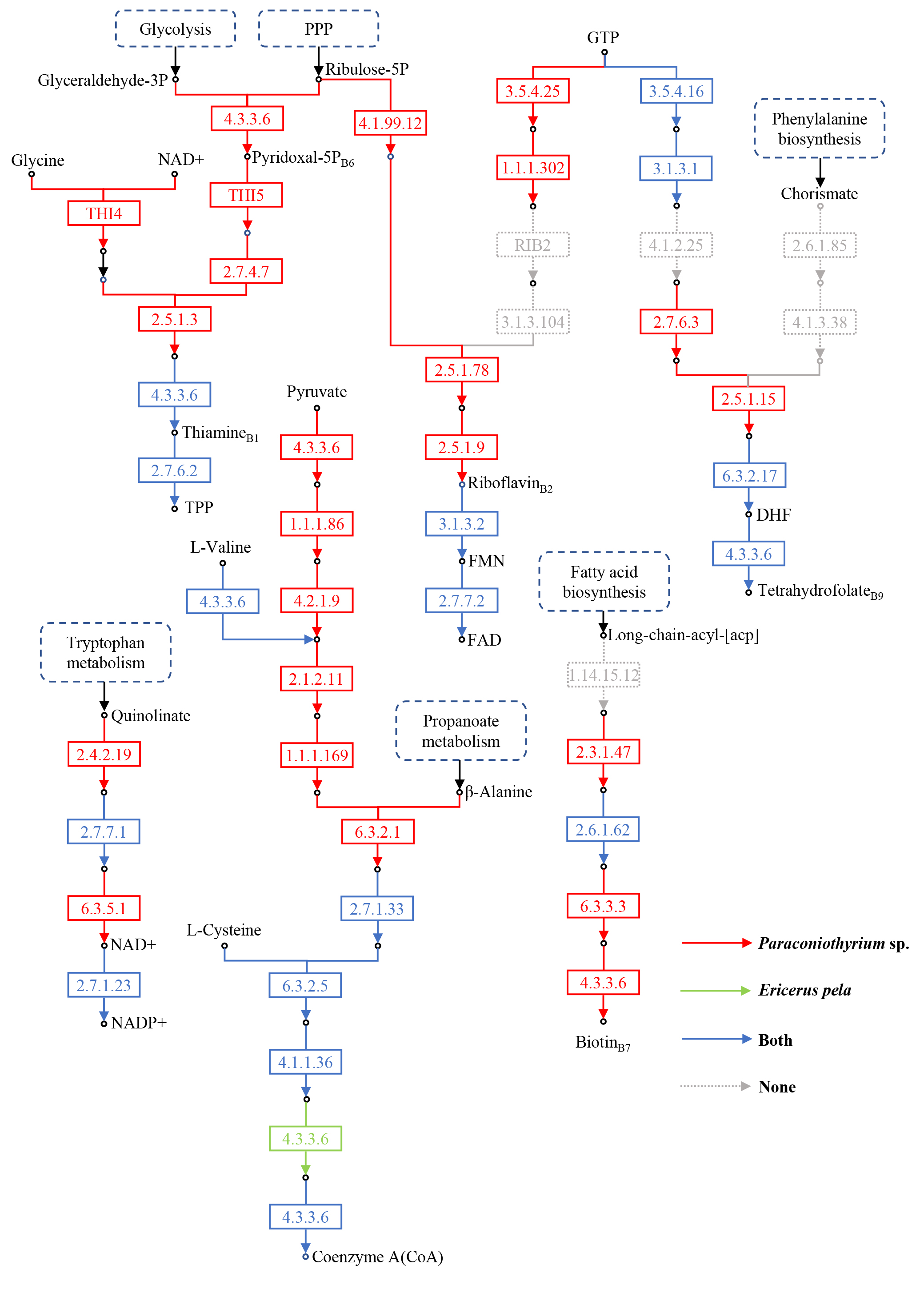

Supplement: Supplementary file 1 [file genes-13-00338-s001.zip › Figure S5.jpg]

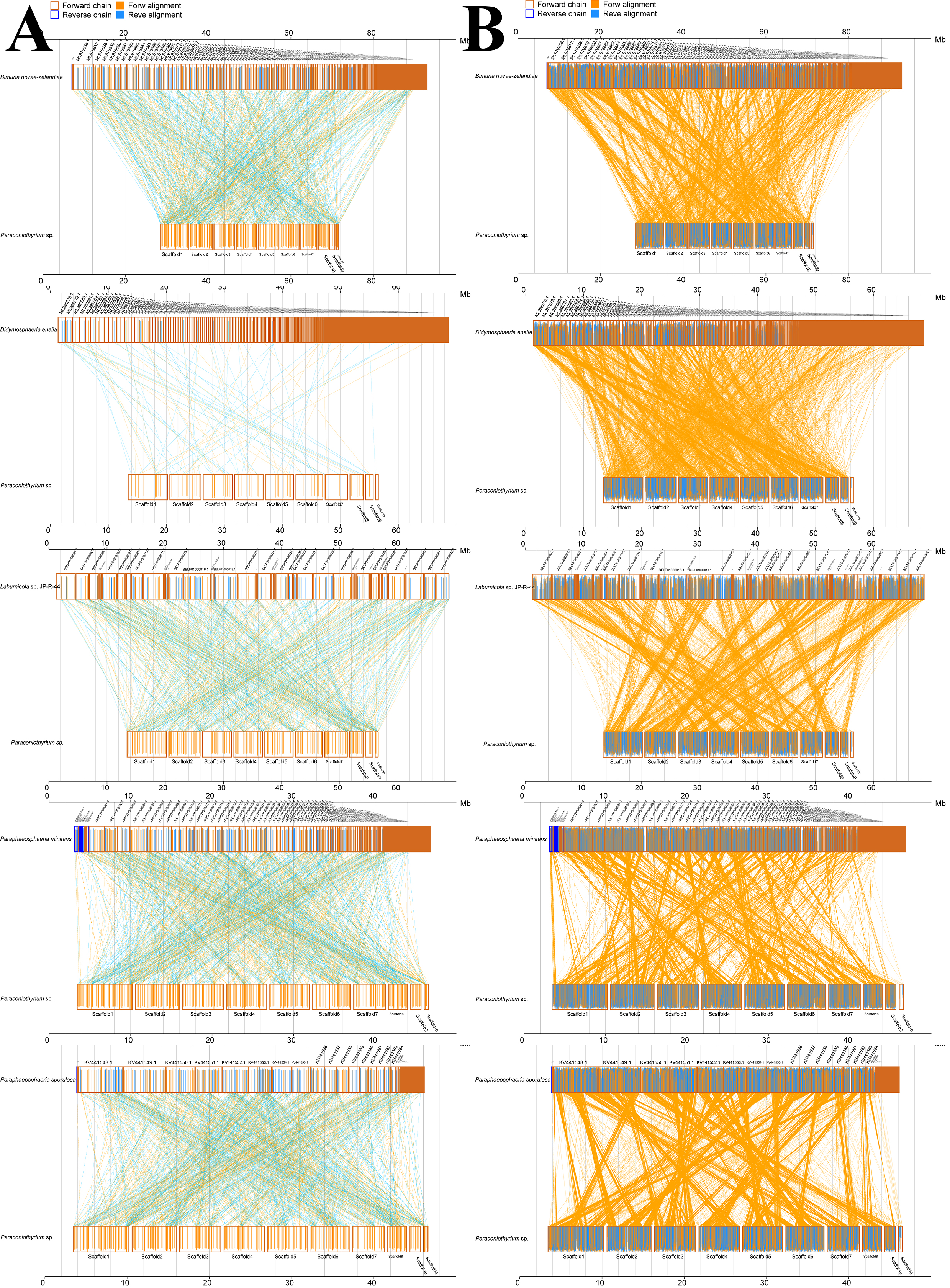

Supplement: Supplementary file 1 [file genes-13-00338-s001.zip › Figure S6.jpg]

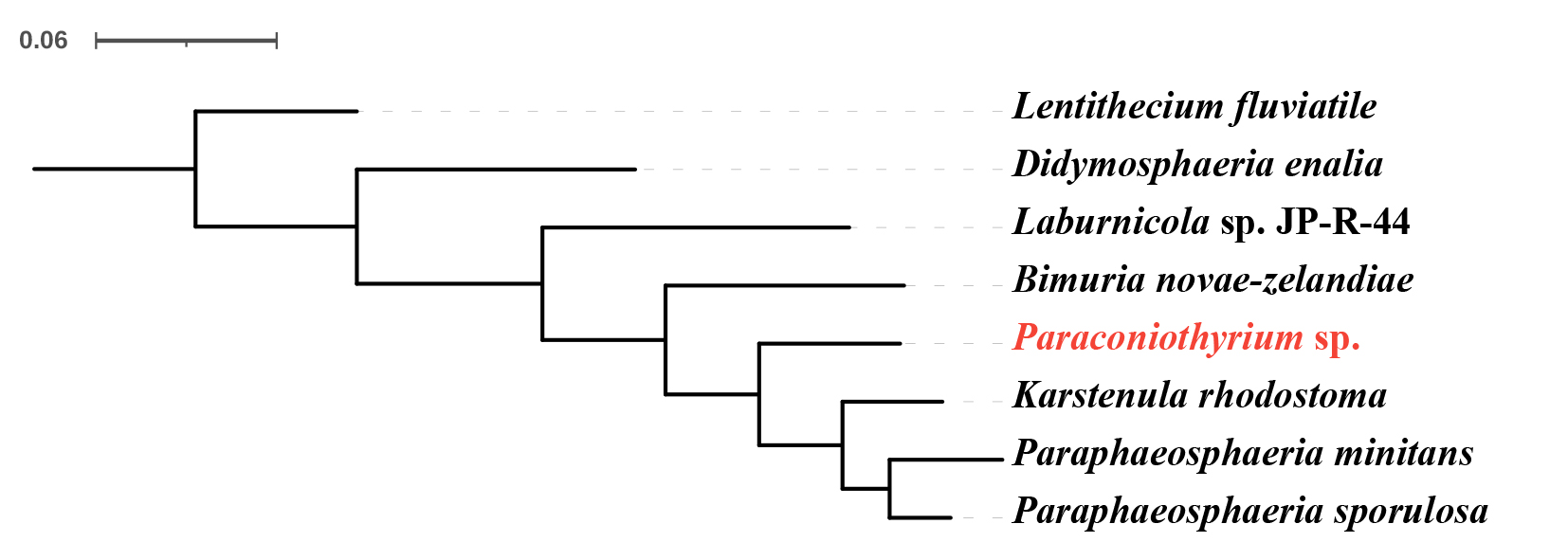

Supplement: Supplementary file 1 [file genes-13-00338-s001.zip › Figure S7a.jpg]

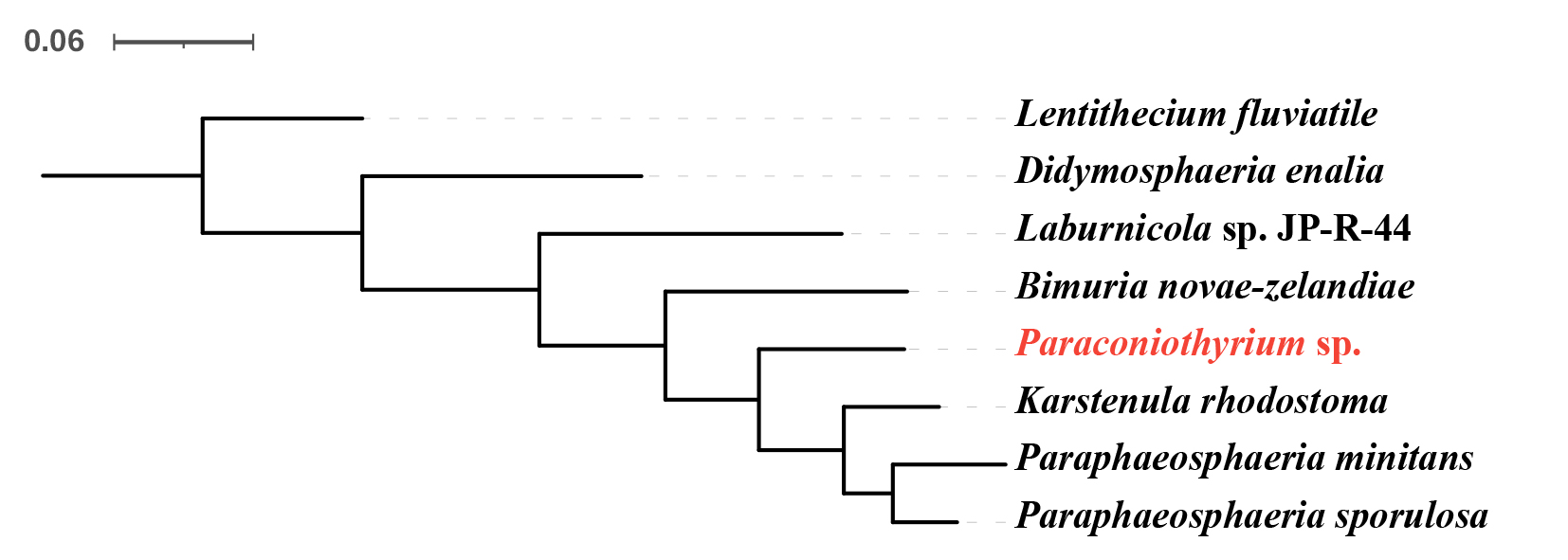

Supplement: Supplementary file 1 [file genes-13-00338-s001.zip › Figure S7b.jpg]
